# Identification of a five-oxidoreductase-gene cluster from *Acetobacter pasteurianus* conferring ethanol-dependent acidification in *Escherichia coli*

**DOI:** 10.1002/mbo3.4

**Published:** 2012-03

**Authors:** Tamara Garcia-Armisen, Ken Vercammen, Tom Rimaux, Gino Vrancken, Luc De Vuyst, Pierre Cornelis

**Affiliations:** 1 Department of Bioengineering Sciences, Research group Microbiology, VIB Department of Structural Biology Vrije Universiteit BrusselPleinlaan 2 1050Brussels Belgium; 2 Research Group of Industrial Microbiology and Food Biotechnology (IMDO), Faculty of Sciences and Bio-engineering Sciences, Vrije Universiteit BrusselBrussels, Belgium

**Keywords:** Central metabolism, gene prediction, gene transfer, hydrogenases

## Abstract

*Acetobacter pasteurianus*, a Gram-negative bacterium belonging to the α-divison of Proteobacteria, produces acetic acid through ethanol oxidation. A genomic bank of *A. pasteurianus* 386B DNA was cloned in the low-copy cosmid pRG930Cm vector and the resulting clones were screened for the production of protease using the skimmed-milk agar assay whereby a clearing zone around the inoculated spots indicates casein degradation. Several positive clones were selected and restriction analysis revealed that many contained the same inserts. One clone was further analyzed and the cosmid DNA subjected to in vitro transposon insertion. After electroporation, several clones having lost the capacity to cause casein degradation were isolated and the sequence of the transposon-flanking regions analyzed. The majority of insertions mapped to one gene encoding an NAD(P)^+^-dependent aldehyde dehydrogenase (ALDH) of the PNTB superfamily, whereas one insert was found upstream in a gene encoding an ethanol dehydrogenase. Addition of phenol red to the medium confirmed the ethanol-dependent acidification around the inoculated spots of the clones without transposon insertion, suggesting that casein degradation is due to the production of acetic acid as a result of the combined activities of the alcohol dehydrogenase and ALDH. Quantitative data and pH measurements confirmed a significant acidification, and the presence of acetic acid.

## Introduction

Acetic acid bacteria (AAB) are Gram-negative α-Proteobacteria that are widely used for vinegar production because of their capacity to oxidize ethanol to acetic acid and to tolerate high concentrations of acetic acid (Kesters et al. 2006; Nakano and Fukaya 2008). Three major genera have been described extensively, namely *Gluconobacter*, *Gluconoacetobacter*, and *Acetobacter*, which are of industrial importance (Yamada et al. 1997; Nakano and Fukaya 2008; Matsutani et al. 2011). *Acetobacter* and *Gluconacetobacter* are the best acetic acid producers and show high acetic acid resistance ability (Matsutani et al. 2011); they are also able to overoxidize acetic acid to carbon dioxide and water (Gullo and Giudici 2008). Further, AAB are key players in the cacao bean fermentation process since the acetic acid they produce kills the plant embryos and because of their contribution to flavor (Camu et al. 2007; De Vuyst et al. 2008; Papalexandratou et al. 2009; Lefeber et al. 2010). One of the most frequently isolated AAB from fermented cacao beans is *Acetobacter pasteurianus* (Camu et al. 2007; Garcia-Armisen et al. 2010). Three genomes of representatives of these AAB genera have been sequenced, one from *Gluconobacter oxydans* (Prust et al. 2005), one from *A. pasteurianus* (Azuma et al. 2009), and one from *Gluconoacetobacter diazotrophicus*, a sugarcane endophytic bacterium (Bertalan et al. 2009).

The present study original aim was to screen for proteolytic enzymes based on the detection of clearing zones of appropriate agar media containing milk casein. For this reason, a genomic library of *A. pasteurianus* was constructed in the wide-host-range low-copy cosmid vector pRG930cm, which has been successfully used in the past for the construction of stable libraries from different *Pseudomonas* strains and from *Burkholderia multivorans* (Lim et al. 1997; Matthijs et al. 2004; Plesa et al. 2006; Chibeu et al. 2009a,2009b). Since the screening for protease activity using skimmed-milk agar plates allows fast and easy detection of extracellular protease activity, we decided to use this procedure to detect clones containing *A. pasteurianus* inserts conferring casein degradation activity evidenced by a clearing zone around the colonies producing a proteolytic enzyme.

However, to our surprise, analysis of the insert followed by in vitro transposon inactivation led to the discovery of the involvement of an alcohol dehydrogenase (ADH) and an aldehyde dehydrogenase (ALDH) gene in the degradation process, via the acidification of the medium due to the release of acetic acid. This is the second time that use of skimmed-milk agar for the detection of protease-producing clones was found to lead to false positives (Jones et al. 2007).

## Results

### Construction of the *A. pasteurianus* genomic library

**Figure 1:**
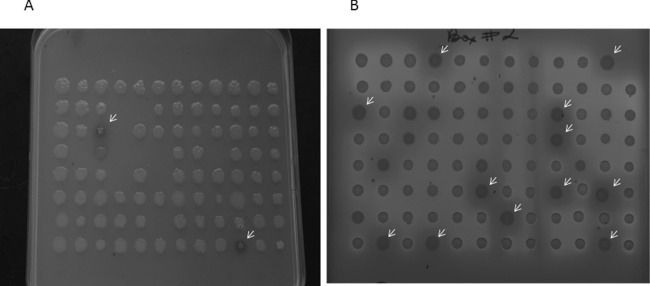
(A) Casein-clearing activity of two cosmid clones containing *Acetobacter pasteurianus* 386B inserts (indicated by arrows). (B) Clone 7C3 cosmid DNA was extracted and submitted to in vitro transposon mutagenesis and the resulting cosmids used to transform *Escherichia coli* by electroporation. Clones having lost the ability to degrade casein are indicated by arrows.

After in vitro packaging and *Escherichia coli* host infection, more than 3000 transformant colonies were obtained. Ten clones were randomly selected and their cosmid DNA was extracted. Restriction analysis revealed that they contained inserts ranging from 20 to 30 kb. The whole library was then replica-plated on Luria Bertani (LB) medium containing skimmed milk and, after overnight incubation, four clones were selected that showed a good clearing zone around the inoculation spot (Fig. 1A). Two clones having an identical restriction pattern showed the highest casein degradation activity and one of these clones (7C3) was selected for further analysis. The 7C3 clone contained an insert of about 25 kb.

### In vitro transposon mutagenesis of the skimmed-milk degrading 7C3 clone

**Figure 2:**
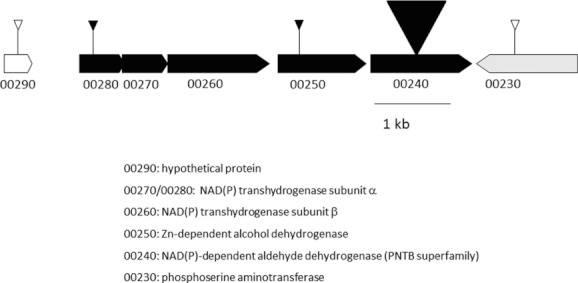
Schematic presentation of the gene cluster conferring casein degradation and medium acidification: the dehydrogenases are shown in black; the transposon insertions that cause an inactivation of these two activities are shown in black, while one transposon insertion that had no effect is shown in white. The numerous transposon insertions in the aldehyde dehydrogenase (ALDH) gene are shown by a large inverted triangle. The proteins encoded by the different genes are mentioned below the figure.

The cosmid DNA from the 7C3 clone was isolated and subjected to in vitro transposon mutagenesis. Clones failing to produce a clearing zone were selected (Fig. 1B) and their cosmid DNA was isolated. Two clones that retained the hydrolytic activity toward casein were also selected as control. Using primers annealing to the transposon, the nucleotide sequences were determined and compared to the NCBI database using the BLASTX algorithm. During the course of this study, the genome sequence of *A. pasteurianus* IFO 3283-01 went public, which greatly facilitated the analysis (Azuma et al. 2009). In total, 6248 nucleotides were sequenced and Fig. 2 shows the 5-kb region and the transposon insertions that caused an absence of skimmed-milk clearing. Almost all insertions leading to disappearance of the skimmed-milk clearing zone mapped to a single gene, APA01_00240 encoding an NAD(P)^+^-dependent ALDH. One insertion was found to be located in the gene upstream of APA01_00240, namely APA01_00250 encoding a Zn-dependent ADH. One insertion inactivating the casein degradation capacity was found in the first gene of the cluster, APA01_00280, which, together with APA01_00270, encodes the α subunit of an NAD(P)^+^ transhydrogenase, the β-subunit being encoded by the downstream gene APA01_00260. These five genes form therefore a cluster of genes, all of which encode oxidoreductases, and which could form an operon. Separated by the APA01_00290 gene, which encodes a LysR regulator, another cluster of five genes, transcribed on the opposite strand, also encodes dehydrogenases with one aspartate semialdehyde encoding gene (APA01_00300) followed by genes encoding the different components of the succinate dehydrogenase complex (results not shown).

### Acidification of the medium is ethanol dependent

**Figure 3:**
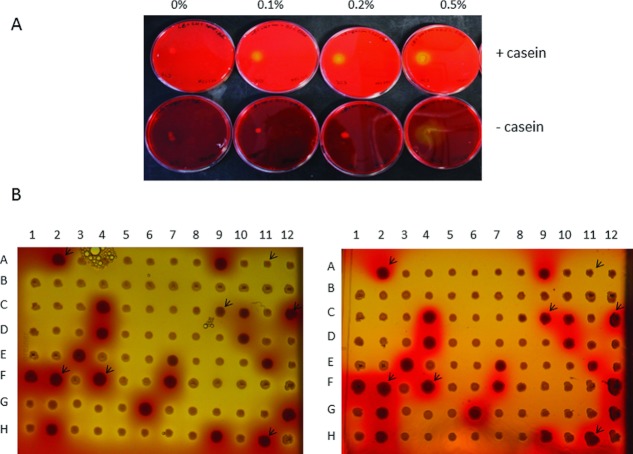
(A) Ethanol-dependent acidification and casein degradation: in the top row are plates containing skim milk (casein), chloramphenicol (Cm) (0.01% ethanol), phenol red, and increasing concentrations of ethanol; in the bottom row are plates containing only Cm and phenol red. (B) Screening of in vitro transposon mutants for medium acidification and casein degradation (left plate) and for medium acidification only (right plate). The clones highlighted with arrows correspond to insertions in the ALDH gene, the locations of which are shown in Figure 4.

Since the in vitro transposon mutagenesis resulted in the inactivation of dehydrogenases, we reasoned that the casein clearance observed when clone 7C3 was plated on medium containing chloramphenicol was due to the conversion of ethanol to acetic acid, since the antibiotic stock solution was made in ethanol, resulting in the presence of the alcohol in the medium (0.01%). To confirm this hypothesis, we plated the 7C3 clone and two clearance-negative clones on media containing spectinomycin (the other antibiotic that can be used to maintain the pRG930-Cm cosmid), since spectimomycin is solubilized in water. According to our expectations, in this case, no clearing of casein was observed (results not shown). When increasing concentrations of ethanol were added to the medium containing chloramphenicol (Cm), clearing of casein was observed as well as a shift of phenol red to yellow, indicating an acidification of the medium, which was maximum for 0.5% ethanol. Already in the presence of 0.01% ethanol due to the presence of Cm, the acidification was clearly seen (Fig. 3A, 0%). Confirming our previous results, the clones with transposon insertions in the ALDH or the ADH did not show such an acidification or casein clearing when ethanol was present in the medium (Fig. 3B).

**Figure 4:**
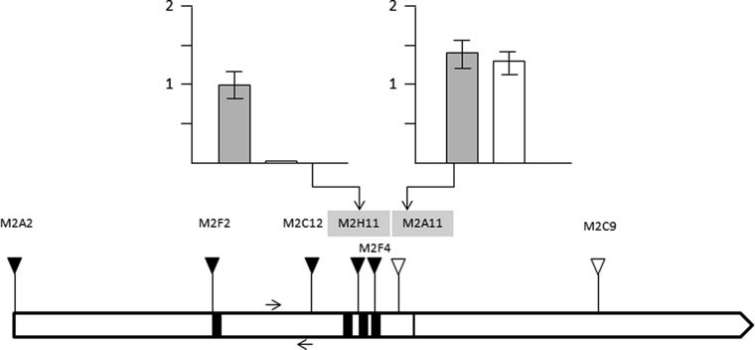
Schematic representation of the ALDH gene showing the places of transposon insertions: insertions causing a phenol red-negative phenotype are indicated by a black triangle and those with a positive phenotype by a white triangle. The residues predicted to be in the active site are shown by black bars. Insert: real-time PCR expression of the alcohol dehydrogenase (ADH) (gray bars) and ALDH (white bars) genes in mutants M2A11 (acidification-positive) and M2H11 (acidification-negative). The ALDH gene was not expressed in M2H11 while the mutation had no effect on the expression of the upstream ADH gene. The arrows represent the positions of the primers used to amplify the ALDH cDNA.

Interestingly, some insertions in the ALDH gene did not entirely eliminate the acidification of the medium as shown in Figure 3B and Fig. 4. These insertions were found to cluster in the second half of the gene, while the mutations causing a total arrest of acidification were all localized in the first half of the ALDH gene. Analysis of the protein sequence revealed that the active sites clustered in this first region, suggesting that the C-terminal portion of the enzyme was not essential for its activity.

### Real-time PCR detection of the expression of the ADH and ALDH genes

The level of expression of the ADH and ALDH genes was measured via real-time PCR. Two transposon mutants were selected, M2A11 (acidification-positive) and M2H11 (acidification-negative) (Fig. 4). The primers were designed to allow amplification of a cDNA fragment corresponding to a region left of the H11 insertion (Figure 4). As shown in Figure 4, in mutant A11, amplification signals were detected for both ADH and ALDH, while, as expected, no signal was observed for ALDH in the H11 mutant. The insertions had therefore no effect on the expression of the ADH gene.

### Detection of acetic acid production

After overnight incubation at 37°C in the presence of 0.1% (v/v) ethanol, the pH of the spent medium was measured and confirmed a significant drop of the pH value for the 7C3 clone and for two positive transposon mutants. Clone C9 (Fig. 3B), which showed an intermediate phenotype (see Fig. 4 for the localization of the transposon insertion), also caused a lower pH drop (Table 1). The pH value of spent medium of acidification-negative clones was between 8.4 and 8.7.

**Table 1:** Summary of the phenotypes (phenol red discoloration, casein degradation, acidification, consumption of ethanol, and acetic acid production) by the *Escherichia coli* clone containing the original cosmid, and by mutants having a transposon inserted in the ALDH gene (Figure 4)

Clone	Phenol red discoloration	Casein degradation	pH of spent medium	Ethanol (mM)	Acetic acid (mM)
7C3	++	++	4.72	27.86 ± 3.44	52.34 ± 1.08
A11	++	++	4.68	22.30 ± 1.46	54.96 ± 3.81
C9	+	+	5.63	40.03 ± 6.88	45.66 ± 4.03
H11	-	-	8.71	ND	37.74 ± 1.93

ND, not done.

## Discussion

**Figure 5:**
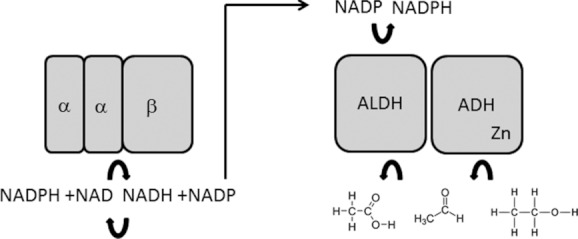
Proposed model of the interaction between the transhydrogenase and the complex between the ADH and the ALDH: the transhydrogenase complex generates NADP^+^ via a transhydrogenation reaction and the Zn^2+^-dependent ADH will oxidize ethanol to acetaldehyde, which can be further oxidized to acetic acid by the ALDH.

AAB are known for their capacity to oxidize ethanol to acetic acid (Nakano and Fukaya 2008). The 2.9-Mb genome of *A. pasteurianus* IFO 3283 has been published recently, revealing the presence of multiple transposon sequences representing 9% of the genome (Azuma et al. 2009). *Acetobacter pasteurianus* is known to produce three different ADHs: one is membrane bound at the periplasmic side and PQQ dependent while the two others are NAD^+^ dependent and localized in the cytoplasm (Chinnawirotpisan et al. 2003a,2003b). It has been shown that a mutant in the PQQ-dependent ADH underwent profound metabolic changes, due to the induction of the cytoplasmic NAD(P)^+^-dependent ADHs, causing a switch from acetic acid production to ethanol assimilation via the TCA and glyoxylate cycles (Chinnawirotpisan et al. 2003b). The ADH gene present in our 5-kb cluster was predicted to encode an NAD(P)^+^- and Zn^2+^-dependent enzyme, corresponding to the previously described ADH1 (Chinnawirotpisan et al. 2003a). Remarkably, the genes for the ADH and ALDH are clustered together in the genome of *A. pasteurianus*, while they are in different loci in the genome of another acetic acid bacterium, *G. oxydans* (Prust et al. 2005). This probably explains why *A. pasteurianus* can oxidize ethanol more efficiently than *Gluconobacter* or *Gluconoacetobacter*. Because the acidification observed was ethanol dependent and clearly absent when one of the two genes (ADH or ALDH) was inactivated, we concluded that the genes were well expressed in *E. coli* and that the following reaction was taking place: ethanol→acetaldehyde→acetic acid, the first reaction being catalyzed by the Zn^2+^-dependent ADH and the conversion from acetaldehyde to acetic acid by the acetaldehyde dehydrogenase. The present study clearly demonstrated that this is the case since acidification of the medium was clearly ethanol dependent. Jones et al. (2007) have also observed the degradation of casein by metagenomic fosmid clones, but, similarly, they failed to detect the involvement of proteases in this process (Jones et al. 2007). Instead, after in vitro transposon mutagenesis, they have found that the genes conferring the clearing of casein encode β-glucosidases, apparently resulting in the degradation of lactose present in skimmed milk, fueling the glycolysis reaction causing the production and release of acetic acid as end product of the fermentation of carbohydrates. In their case, the acidification was an indirect result of the fermentation of lactose, while in our case it was fueled by the ethanol present in the medium. Only the clones with an active ADH and ALDH could cause a significant drop in the pH of spent LB medium. The pH supernatant of cultures of ALDH-negative clones was above 8, in agreement with previously published data showing that LB medium contained no sugars that are fermentable by *E. coli*, explaining why the pH rises in the culture because of deamination of amino acids (Sezonov et al. 2007). Likewise, we found that most of the transposon insertions that caused a casein hydrolysis-negative phenotype also impaired the production of acetic acid. Most of the transposon insertions occurred in the ALDH gene, but some of them still allowed some acidification of the medium, suggesting that a truncated ALDH containing the active sites could still be functional. One transposon insertion causing a clearing-negative phenotype was found to be in the first gene of the cluster encoding the α−subunit of a transhydrogenase. Transhydrogenases catalyze the transfer of protons from NADH + H^+^ to NADP^+^ to form NADPH + H^+^ (Jackson 2003). It is likely that this enzyme is needed for the generation of NADP^+^ for the proper working of the NADP^+^-dependent ALDH (Fig. 5). To the best of our knowledge, this is the first time that the coupled activities of ADH and ALDH have been shown to cause the production of high levels of acetic acid in *E. coli*.

## Experimental Procedures

### Strains and media used

*Acetobacter pasteurianus* 386B, isolated from a heap fermentation in Ghana in 2007 (Camu et al. 2007), was grown in mannitol-yeast extract-peptone medium (MYP) at 28°C under strong agitation. Two *E. coli* strains were used as hosts: VCS257 (Agilent Technologies, Santa Clara, CA), and TransforMax EC100 Electrocompetent (Epicentre Biotechnologies, Madison, WI). *Escherichia coli* strains were grown in LB medium. As plasmid cloning vectors, cosmid pRG930 (Cm′ Sm′ Sp′) was used for the construction of the *A. pasteurianus* genomic library (Matthijs et al. 2004).

Antibiotics were added in the following concentrations: kanamycin (Km), 50 μg/mL; Cm, 20 mg/L, and spectinomycin (Sp), 50 μg/mL.

### Construction and screening of the *A. pasteurianus* genomic library

Cells from an *A. pasteurianus* 386B culture were pelleted and genomic DNA extracted using the Gentra kit for genomic DNA extraction (Qiagen, Hilden, Germany), following the manufacturer's instructions. Genomic DNA was then partially restricted with Bsp143I, using increasing dilutions of this restriction enzyme. After 30 min, the reaction was stopped by incubation at 65°C and 5-μl aliquots from each tube were analyzed by agarose gel electrophoresis. Tubes containing fragments averaging 30 kb were pooled and their DNA ligated to BamH1-restricted cosmid pRG930-Cm vectors (Matthijs et al. 2004; Chibeu et al. 2009b).

Packaging of ligated DNA and transformation of *E. coli* VCS257 was done using a commercially available kit, Gigapack III, following the recommendations of the manufacturer (Agilent Technologies).

Colonies were selected on LB agar with chloramphenicol and spectinomycin, after incubation at 37°C overnight, and transferred with sterile toothpicks to individual wells of 96-well microtitre plates containing 100 μl of the same medium.

The screening for protease activity was performed by replicating the arrayed library on LB agar supplemented with 1% (w/v) skimmed milk and chloramphenicol (SMA).

SMA was prepared as described elsewhere (Jones et al. 2007). Plates were incubated for three days at 37°C, after which any clone with a clear halo was picked up and stored at −80°C in 20% glycerol for subsequent analysis.

### In vitro transposon mutagenesis

Transposon mutagenesis of positive clones was performed using the in vitro EZ::Tn*5* system (Epicentre Biotechnologies). The cosmids containing the transposon were transferred by electroporation into TransforMax EC100 electrocompetent cells (Epicentre Biotechnologies). Clones containing the cosmid and the transposon sequence were selected on LB supplemented with kanamycin, chloramphenicol, and spectinomycin.

All sequencing was performed by the VIB Genomic Service sequencing facilities (Antwerp, Belgium) using primers specific to the inserted transposon.

### Sequence analysis

The sequences obtained were assembled manually and compared with the genome of *A. pasteurianus* IFO 3283-01 in the NCBI database using the BLASTX algorithm (http://www.ncbi.nlm.nih.gov/BLAST). Sequences missing between the sequences flanking the transposon insertions were taken from the *A. pasteurianus* IFO 3283-01 sequence as template (http://www.ncbi.nlm.nih.gov/genomeprj/59279).

### Additional analysis

Additional analysis of the host, the protease-positive clones, and associated mutants was performed on SMA and LB agars, supplemented with 0.25 mg/mL of phenol red as an indicator of acidic pH and with either spectinomycin or chloramphenicol.

These media were supplemented with ethanol at four different concentrations: 0.1%, 0.2%, 0.5%, and 1% (v/v). Media without ethanol were used as control.

### Real-time PCR amplification (RT-qPCR)

Five-milliliter cultures of a positive and negative transposon mutant were grown overnight at 225 rpm at 37°C in LB medium containing 100 mg/L of spectinomycin and 50 mg/L of kanamycin. RNA was extracted from 0.5-mL cultures using the RNeasy Mini kit (Qiagen) and following the manufacturer's instructions, except for the DNAse treatment. Briefly, DNA was removed from 30-μg RNA/DNA samples using recombinant DNAse I (ROCHE, Indianapolis, IN) and according to the manufacturer's instructions.

cDNA was made from 200-ng RNA using the ProtoScript® M-MuLV First Strand cDNA Synthesis Kit (New England Biolabs, Massachusetts, MA).

RT-qPCR was performed using a MyiQ™2 two-color Real-Time PCR detection System (Bio-Rad, Hercules, CA). Each sample was done in triplicate. The ALDH (00240) and ADH (00250) genes were amplified in a total volume of 25 μl containing 12.5 μl of iQ™ SYBR Green Super Mix (Bio-Rad), 1 μl of cDNA template (50 ng), and 0.2 μM of each primer. The following primers were used for the amplification of the ADH gene (ALD-FW 5′-GTAGCAATGGGGCTGAATGT-3′ and ALD-REV 5′-CCACCAACTTTTTCCTGCAT-3′) and the ALDH gene (ALDH-FW 5′-GCCATGGAAAAACTGATGCT-3′ and ALDH-REV 5′-ACGCGTGGGTCTTGAATAAC-3′).

The PCR thermocycling steps were set as follows: 95°C for 5 min and 40 cycles at 95°C for 30 sec and 55°C for 30 sec. The negative control containing no DNA was subjected to the same procedure to exclude or detect any possible contamination. After the RT-qPCR assay, the specificity of amplification was verified by melting curve analysis.

### Detection of ethanol and acetic acid

The concentrations of ethanol and acetic acid in the culture media was measured by gas chromatography (GC), using a Focus gas chromatograph (Interscience, Breda, the Netherlands), equipped with a Stabilwax-DA column (Restek, Bellefonte, PA), a flame ionization detector (FID), and an AS 3000 autosampler, as described previously (Lefeber et al. 2010). Quantifications of the ethanol and acetic acid concentrations was performed using external standards. Samples were analyzed in triplicate and the results are represented as the average of these three independent measurements. The errors on the measurements are represented as standard deviations.
